# Interannual Variation of the Bowen Ratio in a Subtropical Coniferous Plantation in Southeast China, 2003-2012

**DOI:** 10.1371/journal.pone.0088267

**Published:** 2014-02-10

**Authors:** Yakun Tang, Xuefa Wen, Xiaomin Sun, Huimin Wang

**Affiliations:** 1 Key Laboratory of Ecosystem Network Observation and Modeling, Institute of Geographic Sciences and Natural Resources Research, Chinese Academy of Sciences, Beijing, China; 2 University of Chinese Academy of Sciences, Beijing, China; The Ohio State University, United States of America

## Abstract

The interannual variation of the Bowen ratio, through its effect on the warming extent of available energy to the ecosystem land surface air, heavily influences the ecosystem microclimate and affects the hydrological cycle at both regional and global scales. Although the precipitation amount in southeast China is not expected to change greatly as a result of climate change, the precipitation frequency may be altered in the future. We explored the interannual variation of the Bowen ratio and its affecting mechanisms based on eddy covariance measurements in a subtropical plantation in southeast China during 2003–2012. The results indicated that the annual mean Bowen ratio was 0.35±0.06, with a range of 0.29–0.45. The Bowen ratio during the dry season (July-October) positively correlated with the annual Bowen ratio (R^2^ = 0.85, p<0.001). The effective precipitation frequency during the dry season, through its positive effect on shallow soil water content, indirectly and negatively affected the annual Bowen ratio. Between 2003 and 2012, the annual Bowen ratio exhibited a marginally significant decreasing trend (p = 0.061), meanwhile the effective precipitation frequency and shallow soil water content during the dry season increased significantly (p<0.001). The annual Bowen ratio may decrease further if the effective precipitation frequency and shallow soil water content during the dry season follow similar trends in the future. The warming effect of available energy to the surface air of our studied plantation may decline with the decreasing annual Bowen ratio.

## Introduction

The consumption of available energy as sensible heat flux warms the land surface air [1]. While energy consumption as latent heat flux, which is equivalent to evapotranspiration and responsible for a big part of precipitation in many parts of the world, mitigates this warming effect [1], [2]. The Bowen ratio, which is the ratio of the sensible heat flux to latent heat flux, through its effect on the warming extent of available energy to the ecosystem land surface air, heavily influences the ecosystem microclimate and the hydrological cycle at both regional and global scales [3]–[5].

Forests cover approximately 31% of the total global land area [6] and are vital components of energy cycles between the terrestrial surface and atmosphere [4], [7]. Numerous models have suggested that the Bowen ratio will decline in the future because the increased energy due to carbon dioxide (CO_2_) is primarily consumed as latent heat flux rather than as sensible heat flux [8], [9]. However, diverse long-term trends of the annual Bowen ratio that measured directly by the eddy covariance method have been observed among forest ecosystems [10]–[13]. Keenan et al. [10] demonstrated that the Bowen ratio shows a global increasing trend due to the adjustment of leaf stomata in response to increasing atmospheric CO_2_, based on the energy fluxes of 21 forest sites ranging from 7 to 18 years. While the annual Bowen ratio shows a decreasing trend in a boreal Scots pine forest [11]. And no significant trend is observed in a broadleaved forest [3], in a temperate spruce forest [12], or in a Japanese cypress forest [13]. There is a lack of consensus between these models and the observations because these models do not fully considering the interactive relationships between the atmosphere-biosphere energy fluxes and vegetation in response to climate change, especially for forests [8], [14].

Because the forest Bowen ratio is influenced by the interactive effects of environmental and physiological factors, the dominant factors affecting the variation of the Bowen ratio are also diverse [2], [4], [7]. Wilson et al. [7] demonstrated that physiological factor (vegetation surface resistance) dominate the discrepancy of the Bowen ratio among different forest types in temperate regions. While, environmental factors are shown to primarily affect the discrepancy of the Bowen ratio among forest types in East Asia [2]. For a specific forest, the interannual variation of the Bowen ratio in a boreal Scots pine forest [11] is primarily attributed to net radiation, and the precipitation amount dominates the interannual variation of the Bowen ratio in a temperate spruce forest [12]. In addition, seasonal precipitation affects forest ecosystem energy fluxes in a nonlinear fashion [15]–[17]. There is a need to recognize the influence of the seasonal distribution of precipitation on the interannual variation of the Bowen ratio [17].

Coniferous forests account for approximately 36% of all global forested areas and are predominantly distributed in subtropical, temperate, and boreal regions [6]. Southern China has the largest global evergreen subtropical forest (covering 53 million ha), and coniferous plantations account for 41% of the total forested area in south China [18]. Although the precipitation amount in southeast China is not expected to change significantly because of climate change, the precipitation frequency is expected to change in the future [19]. This region is characterized by a subtropical humid monsoon climate with abundant water and energy resources [20], while drought stress occurs during July-October as a result of different distributions of temperature and precipitation [21]. In southeast China, previous studies in a subtropical coniferous plantation at the Qianyanzhou site mainly focus on the effect of drought stress on the interannual variation of the carbon exchange process [20], [22]. However, the interannual variation of the Bowen ratio in response to drought stress has yet to be investigated. In this study, we examined the energy fluxes during 2003–2012 at the Qianyanzhou site. The objectives of this study were (1) to investigate the interannual variation of the Bowen ratio and its affecting mechanism, (2) to identify the seasonal drought effect on the interannual variation of the Bowen ratio, (3) to study and analyze the long-term trend of annual Bowen ratio.

## Materials and Methods

### Ethics Statement

The study site is maintained by the Institute of Geographic Sciences and Natural Resources Research, Chinese Academy of Sciences. This area is a practice base for researchers at the Chinese Academy of Sciences. All necessary permits were obtained for this field study. The field study did not involve endangered or protected species.

### Site Description

The Qianyanzhou site (26°44′52″N, 115°03′47″E, and elevation 102 m) is a member of ChinaFLUX and is located at the Qianyanzhou station of the Chinese Ecosystem Research Network (CERN) in southeast China. This area is influenced by a subtropical monsoon climate, where the annual mean temperature and precipitation are 17.9 °C and 1472.8 mm, respectively, according to meteorological records of 1985–2012. The soil is red earth, largely weathered from red sandstone, and is classified as Typic Dystrudepts Udepts Inceptisols according to the United States soil taxonomy [21]. The soil texture is divided into the following particle grades: 2.0–0.05 mm (17%), 0.05–0.002 mm (68%), and <0.002 mm (15%) [20]. The soil bulk density at the surface (0–40 cm) is 1.57 g cm^−3^.

The evergreen coniferous plantation was planted in 1985, and the dominant tree species are Masson pine (*Pinus massoniana L.*), Slash pine (*Pinus elliottii E.*), and Chinese fir (*Cunninghamia lanceolata L.*). According to a survey conducted in 2008, the stem densities of Masson pine, Slash pine, and Chinese fir were 700, 545, and 93 stems ha^−1^, respectively; the mean heights were 11.2, 14.3, and 11.8 m, respectively; and the mean diameters at the breast height were 13.6, 18.2, and 13.8 cm, respectively. Further characteristics of the site are provided in Wen et al. [20] and Zhang et al. [23].

### Eddy Covariance and Meteorological Measurements

The eddy covariance instruments were mounted at 39.6 m on a tower. The concentrations of carbon dioxide (CO_2_) and water vapor were measured using an LI-7500 open-path CO_2_/H_2_O analyzer (Model LI-7500, Licor Inc., USA), and the three-dimensional wind speed and virtual temperature were detected by a three-dimensional sonic anemometer (Model CSAT3, Campbell Scientific Inc., USA). All raw data were sampled at 10 Hz, and the 30 min mean fluxes were calculated and stored by a CR5000 datalogger (Campbell Scientific Inc., USA).

A suite of meteorological measurements was also measured at the site. A four-component net radiometer (Model CNR-1, Kipp & Zonen Inc., The Netherlands), a quantum sensor of photosynthetically active radiation (Model LI190SB, Licor Inc., USA), and a pyranometer (Model CM11, Kipp & Zonen Inc., The Netherlands) were used to measure radiation. Air temperature and relative humidity sensors (Model HMP45C, Campbell Scientific Inc., USA) and wind speed sensors (A100R, Vector Inc., USA) were mounted at heights of 1.6, 7.6, 11.6, 15.6, 23.6, 31.6, and 39.6 m above the ground. Soil temperatures were made at depths of 2, 5, 20, 50, and 100 cm with thermocouples (105T and 107-L, Campbell Scientific Inc., USA). Soil volumetric water contents were recorded at depths of 5, 20, and 50 cm using three TDR probes (Model CS615-L, Campbell Scientific Inc., USA). Two soil heat flux plates (Model HFT-3, Campbell Scientific Inc., USA) were placed at a depth of 5 cm below the ground surface. Precipitation was monitored with a rain gauge (Model 52203, RM Young Inc., USA). All data were collected by three CR10X dataloggers (Campbell Scientific Inc., USA) and a CR23X datalogger (Campbell Scientific Inc., USA) with a 25-channel solid-state multiplexer (Model AM25T, Campbell Scientific Inc., USA). Meteorological factors including solar radiation, net radiation, and precipitation have been observed at a meteorological station less than 1 km from the flux site since 1985. Additional details on data acquisition are described further in our previous studies [20], [21].

### Flux Correction and Gap Filling

Latent heat flux (LE) and sensible heat flux (H) (W m^−2^ s^−1^) between the forest and atmosphere over 30 min intervals were calculated as follows:
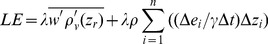
(1)

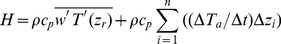
(2)where the first term on right-hand side is the eddy flux of latent heat flux or sensible heat flux, the second term is the storage of latent heat flux or sensible heat flux below the height of observation (*z_r_*), and all advective terms in the mass conservation equation are ignored. The latent heat flux storage term was calculated based on the profile of air temperature and relative humidity, as suggested by McCaughey [24] and Oliphant et al. [25]. The sensible heat flux storage term was computed using the change in the air temperature profile according to Oliphant et al. [25].

Planar fit rotation was applied to remove the effect of instrument tilt or irregularity on the airflow at monthly intervals [20], [26]. The Webb-Pearman-Leuning (WPL) correction was performed to adjust density changes resulting from fluctuations in heat and water vapor [27]. We screened and eliminated the eddy covariance flux data for anomalous or spurious values caused by precipitation, water condensation, and system failure. Any value that exceeded 5 standard deviations in a window of 10 values was deleted. To avoid the possible underestimation of the fluxes under stable conditions during the night, the effect of friction velocity was identified for each year according to the method described by Reichstein et al. [28]. The latent heat flux and sensible heat flux at night (solar elevation angle<0) were excluded when the friction velocity was less than 0.19 m s^−1^, which was the maximum friction velocity threshold among 2003–2012.

Several strategies were adopted to fill the meteorological data and energy flux gaps. First, gaps of meteorological data that were less than 2 h were interpolated linearly from adjacent data points. Longer gaps of solar radiation and net radiation were filled based on the linear regression between these data observed at the flux site and at the meteorological station less than 1 km from the flux site. The regression was calculated at bi-monthly intervals. Longer gaps of air temperature and relative humidity were interpolated from adjacent levels in the measurement profile. Meanwhile, longer gaps of soil water content were filled using the mean diurnal variation method [29]. The annual short and longer data gaps coverage for all meteorological data were no more than 0.6% and 3.1% during 2003–2012, respectively. Then, we filled short (<2 h) latent heat flux and sensible heat flux data gaps using linear interpolation and longer gaps using a marginal distribution sampling method [28]. From 2003 to 2012, the annual mean short gaps coverage for latent and sensible heat fluxes were 1.6% and 1.4%, respectively. The annual mean daytime longer gaps coverage for latent and sensible heat fluxes were 19.1% and 19.2%, respectively. Meanwhile, the annual mean nighttime longer gaps coverage for latent (79.3%) and sensible heat (80.1%) fluxes were larger than that in daytime, respectively, due to the nighttime data filtration through the friction velocity.

The energy balance ratio (*EBR*) was used to assess the performance of the eddy covariance system. The ratio was calculated from 30 min mean available values for each year as [30]: 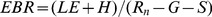
(3)where *R_n_* is the net radiation, *G* is the soil heat flux, and *S* is the heat storage below the observation height.

The energy balance ratio in this study ranged from 0.59 (2005) to 0.76 (2009), which fell within the range of 0.34 to 1.69 reported at FLUXNET sites [30].

### Bulk Canopy Parameters and Potential Evapotranspiration

Daily canopy conductance (g*_c_*) was calculated by averaging 30 min mean values from 10:00 to 16:00 of the Chinese Standard Time (available data>80%). Data on rainy days were omitted to avoid wet canopy. Canopy conductance is sensitive to drought stress [5] and was computed by inverting the Penman-Monteith equation [31], [32],
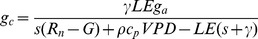
(4)

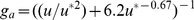
(5)where *s* is the slope of a function relating saturation vapor pressure to temperature (kPa K^−1^), *γ* is the psychrometric constant (kPa K^−1^), *LE* is the latent heat flux (W m^−2^), *C_p_* is the specific heat of air (J kg^−1^ K^−1^), *VPD* is the air vapor pressure deficit (kPa), *ρ* is the air density (kg m^−3^), *g_a_* is the aerodynamic conductance (mm s^−1^), *u* is the wind speed (m s^−1^), and u* is the friction velocity (m s^−1^).

The Priestley-Taylor (*α*) is the ratio of the measured evapotranspiration (ET) to the equilibrium evapotranspiration (ET_eq_). The equilibrium evapotranspiration is considered the climatologically expected evapotranspiration, which assumes a closed volume with constant energy input over a ‘wet’ surface [33]. A small value for Priestley-Taylor (*α*) is indicative of a lack of water supply for evapotranspiration [5], [34]. Potential evapotranspiration was calculated as 1.26 × the equilibrium evapotranspiration [35].
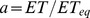
(6)

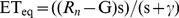
(7)


### Budyko’s Aridity Index

Budyko’s aridity index is the ratio of the precipitation amount to potential evapotranspiration [36]. When Budyko’s aridity index is smaller than one, the system is water limited. This criterion has been used to detect periods of drought stress in coniferous forest and grassland ecosystems [34], [37]. Ryu et al. [34] also suggested that a seasonal timescale is appropriate for indicating the drought stress period, especially considering the influence of vegetation.

### Remotely Sensed Enhanced Vegetation Index

The enhanced vegetation index (EVI) reflects both the leaf area and the canopy biophysical characteristics [38]. This index is resistant to the soil background and less susceptible to atmospheric disturbance [38], [39], and has been considered a physiological affecting factor to the net ecosystem production for evergreen coniferous and broadleaved deciduous forests [25], [40]. The 8-day enhanced vegetation index at a spatial resolution of 500 m was calculated according to Huete et al. [38]. The MODIS/Terra Surface Reflectance 8-Day L3 Global 500 m (MOD09A1) data were obtained from the data portal of the Earth Observation and Modeling Facility at the University of Oklahoma. The Savitzky-Golay method was adopted to detect and replace abnormal enhanced vegetation index values [23], and a window length parameter of 4 was used in this method.

### Effective Precipitation Frequency

The effective precipitation frequency of the studied plantation was defined as the number of days during a specific period when the daily precipitation amount exceeded 1.5 mm, as suggested by Wang et al. [21]. This criterion was chosen because it is the minimum daily precipitation amount that can influence the shallow soil water content in this studied plantation.

### Data Statistics Analysis Method

In this study, the monthly and annual latent and sensible heat fluxes were referred to the sum of these fluxes at each timescale. The monthly and annual Bowen ratio was calculated as a ratio of the sensible heat flux to the latent heat flux at each timescale. Baldocchi et al. [41] suggested that monthly timescale is a pronounced spectral gap in an ecosystem to capture seasonal change of energy fluxes. The independent factors (environmental or physiological) that affect the annual Bowen ratio can be used to explain the interannual variation of the Bowen ratio. In this study, through a single factor regression analysis between the independent factors and the annual Bowen ratio, the dominant affecting factor for the interannual variation of the Bowen ratio was detected. The environmental factors include net radiation, air temperature, vapor pressure deficit, soil water content at three depths (5, 20, and 50 cm) and precipitation amount. The physiological factor is referred to the enhanced vegetation index.

For the single factor regression analysis, the coefficient of determination (R^2^) and *p*-value were reported. First, for each timescale, the dominant affecting factor for the Bowen ratio was selected based on the maximum coefficient of determination. The residual between the Bowen ratio and the fitted Bowen ratio, which is a function of the dominant factor, was also calculated for each timescale. Then, the regression analysis was performed between other independent factors and the residual. The dominant affecting factor for the residual was also selected based on the maximum coefficient of determination [42]. All statistical analyses were performed using SPSS 13.0.

## Results

### Interannual Variation of Environmental and Physiological Factors

**Figure 1:**
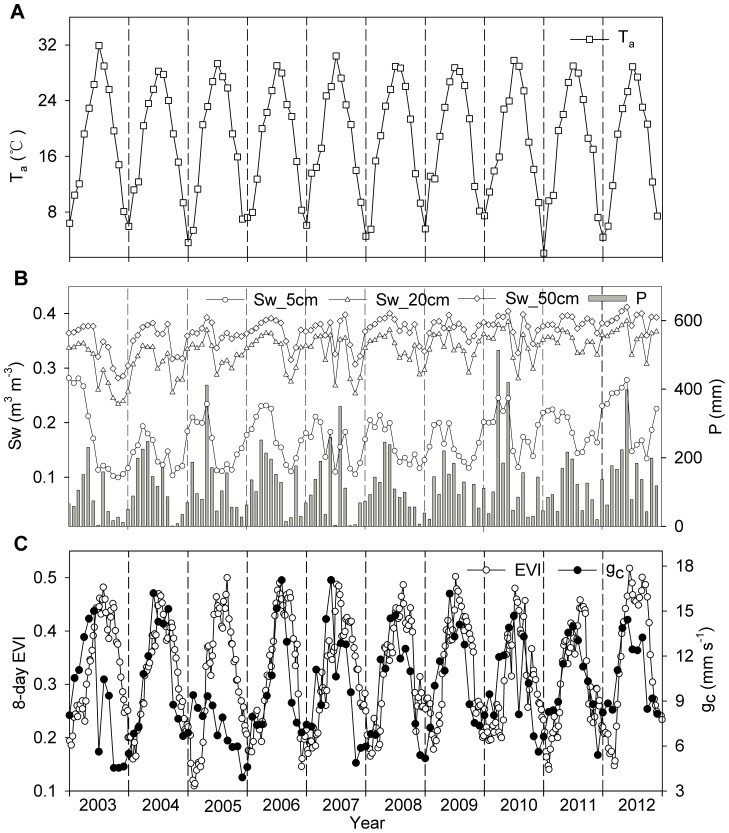
Seasonal and interannual variation of environmental and physiological factors. Factors include (A) monthly mean air temperature (T_a_), (B) monthly mean soil water content at 5, 20, and 50 cm (Sw_5 cm, Sw_20 cm, and Sw_50 cm) and monthly precipitation amount (P), and (C) monthly mean canopy conductance (g_c_) and 8-day timescale enhanced vegetation index (EVI).

Seasonal variation of monthly environmental factors, canopy conductance (*g_c_*), and the 8-day enhanced vegetation index (EVI) during 2003–2012 are plotted in Fig. 1. The air temperature exhibited a single peak, with the maximum and minimum values occurring in summer (June-August) and winter (December-February), respectively. In general, precipitation experienced more frequently in the first half of the year than in the second half of the year, and precipitation deficiency often occurred in summer. This high temperature and a lack of sufficient precipitation in summer may result in seasonal drought stress in this studied plantation [20]. Seasonal variation of the soil water content at 5, 20, and 50 cm were closely related to the precipitation variability (Fig. 1B). The magnitude of the seasonal variation in soil water content decreased with the increasing soil depth. Generally, canopy conductance exhibited the maximum value in summer (Fig. 1C), while, canopy conductance declined during the summers of 2003, 2007, and 2010. Except in 2005, the seasonal variation of the enhanced vegetation index followed that of canopy conductance. During the summers of 2003, 2007, and 2010, the decreasing magnitude of the enhanced vegetation index was smaller than that of the canopy conductance.

Table 1 lists the annual environmental and enhanced vegetation index values. Except in 2006 and 2010, the precipitation amounts in other years were below the long-term average value (1985–2012, 1472.8 mm). Meanwhile, only the air temperature in 2012 was below its long-term average value (1985–2012, 17.9°C). The maximum and minimum coefficients of variation (CV) of environmental factors were observed in the precipitation amount (20%) and air temperature (3%), respectively.

**Table 1:** Interannual variation of annual net radiation (Rn, MJ m^−2^ yr^−1^), air temperature (T_a_, °C), vapor pressure deficit (VPD, kPa), soil water content at 5 cm, 20 cm, and 50 cm (Sw_5 cm, Sw_20 cm, and Sw_50 cm (m^3^ m^−3^)), enhanced vegetation index (EVI), total precipitation amount (P, mm), latent heat flux (LE, MJ m^−2^ yr^−1^), sensible heat flux (H, MJ m^−2^ yr^−1^), and the Bowen ratio (β) during 2003–2012 at the Qianyanzhou site.

Year	Rn	T_a_	VPD	Sw_5 cm	Sw_20 cm	Sw_50 cm	EVI	P	LE	H	β
2003	2849.3	18.9	0.62	0.18	0.30	0.34	0.34	944.9	1864.8	845.5	0.45
2004	2871.2	18.6	0.54	0.14	0.30	0.35	0.32	1375.5	2061.7	704.4	0.34
2005	2620.8	17.9	0.46	0.16	0.33	0.36	0.30	1455.4	1253.1	564.7	0.45
2006	2658.1	18.4	0.46	0.18	0.33	0.37	0.32	1485.3	1732.5	568.8	0.33
2007	2776.6	18.9	0.52	0.16	0.32	0.36	0.33	1318.7	2125.5	631.8	0.30
2008	2861.1	18.4	0.53	0.16	0.34	0.38	0.33	1332.9	2068.9	768.9	0.37
2009	2939.0	18.7	0.55	0.16	0.34	0.37	0.33	1253.9	2175.3	721.8	0.33
2010	2670.1	18.4	0.51	0.19	0.35	0.38	0.31	1854.3	1911.2	581.9	0.30
2011	2776.5	17.9	0.51	0.19	0.35	0.38	0.30	1237.3	1882.4	547.7	0.29
2012	2725.9	17.4	0.40	0.21	0.36	0.39	0.34	1921.9	1745.0	610.3	0.35
Mean	2774.9	18.4	0.51	0.17	0.33	0.37	0.32	1418.0	1882.0	654.6	0.35
SD	105.38	0.48	0.06	0.02	0.02	0.02	0.01	289.16	268.80	100.63	0.06
CV	0.04	0.03	0.12	0.12	0.06	0.04	0.04	0.20	0.14	0.15	0.17

SD is standard deviation. CV is the coefficient of variation (ratio of standard deviation to mean value).

**Figure 2:**
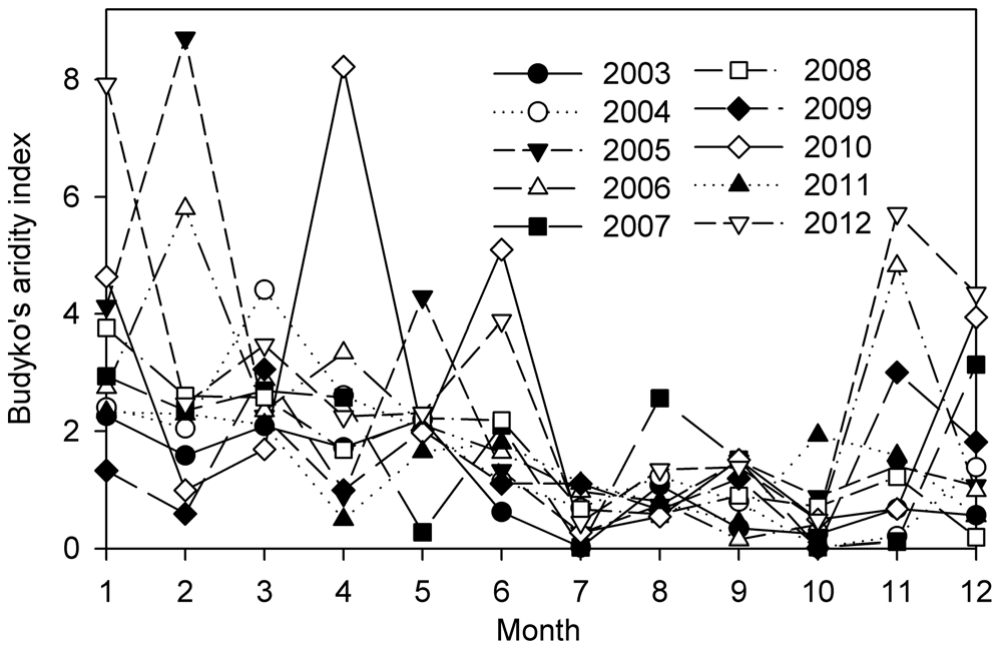
Seasonal and interannual variation of the monthly Budyko’s aridity index.

According to the seasonal variation of Budyko’s aridity index during 2003–2012, the dry season was defined from July to October, and the wet season spanned the remainder of the year (Fig. 2). Wang et al. [21] defined the same period for the dry season in this studied plantation. The annual mean (mean ± one standard deviation) Budyko’s aridity index during the dry season was 0.75±0.17 and ranged from 0.43 (2003) to 0.99 (2007). During the wet season, the Budyko’s aridity index ranged from 1.46 (2003) to 3.45 (2012), with a mean value of 2.22±0.76. The annual mean Priestley-Taylor (*α*) during the dry season (0.58±0.09) was lower than that during the wet season (0.66±0.09), which also indicated the impact of drought stress during the dry season.

### Interannual Variation of the Bowen Ratio

**Figure 3:**
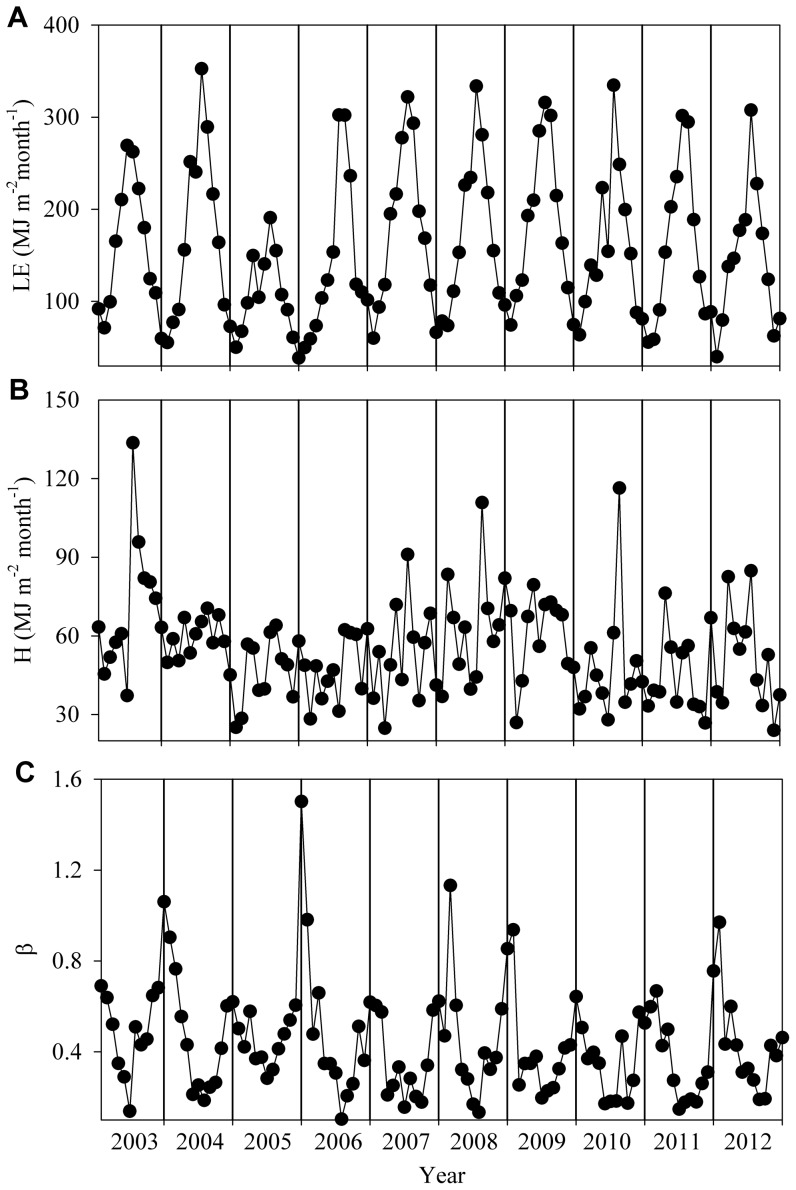
Seasonal and interannual variation of the monthly energy fluxes. Energy fluxes include (A) monthly sum of latent heat flux (LE), (B) monthly sum of sensible heat flux (H), and (C) monthly Bowen ratio (β).

Fig. 3 shows the monthly sum of latent heat flux, monthly sum of sensible heat flux and monthly Bowen ratio. The latent heat flux increased from a minimum during winter to a maximum during summer. The monthly averaged sensible heat flux also showed a similar seasonal variation as latent heat flux, although there was no obviously seasonal variation in 2004. The seasonal variation of the Bowen ratio was ‘U’ shaped, as the latent heat flux overwhelmed the sensible heat flux in almost all months, particularly in summer (Fig. 3C). The Bowen ratio began to decline in winter and the lowest value occurred in summer. However,the Bowen ratio obviously increased during the summers of 2003, 2007, and 2010 because of severe drought stress (Fig. 3C).

The annual means and interannual variation of the latent heat flux, sensible heat flux, and Bowen ratio are listed in Table 1. The annual means of the latent heat flux, sensible heat flux, and Bowen ratio were 1882±269 MJ m^−2^ yr^−1^, 655±101 MJ m^−2^ yr^−1^, and 0.35±0.06, respectively. The maximum values (0.45) of the Bowen ratio were observed in 2003 and 2005, which correspond to a maximum sensible heat flux in 2003 and a minimum latent heat flux in 2005, respectively. Meanwhile, the minimum Bowen ratio (0.29) was observed in 2011 and corresponds to a minimum sensible heat flux. The coefficient of variation of the annual Bowen ratio reached up to 17%, which was higher than those of the latent heat flux (14%) and sensible heat flux (15%).

**Figure 4:**
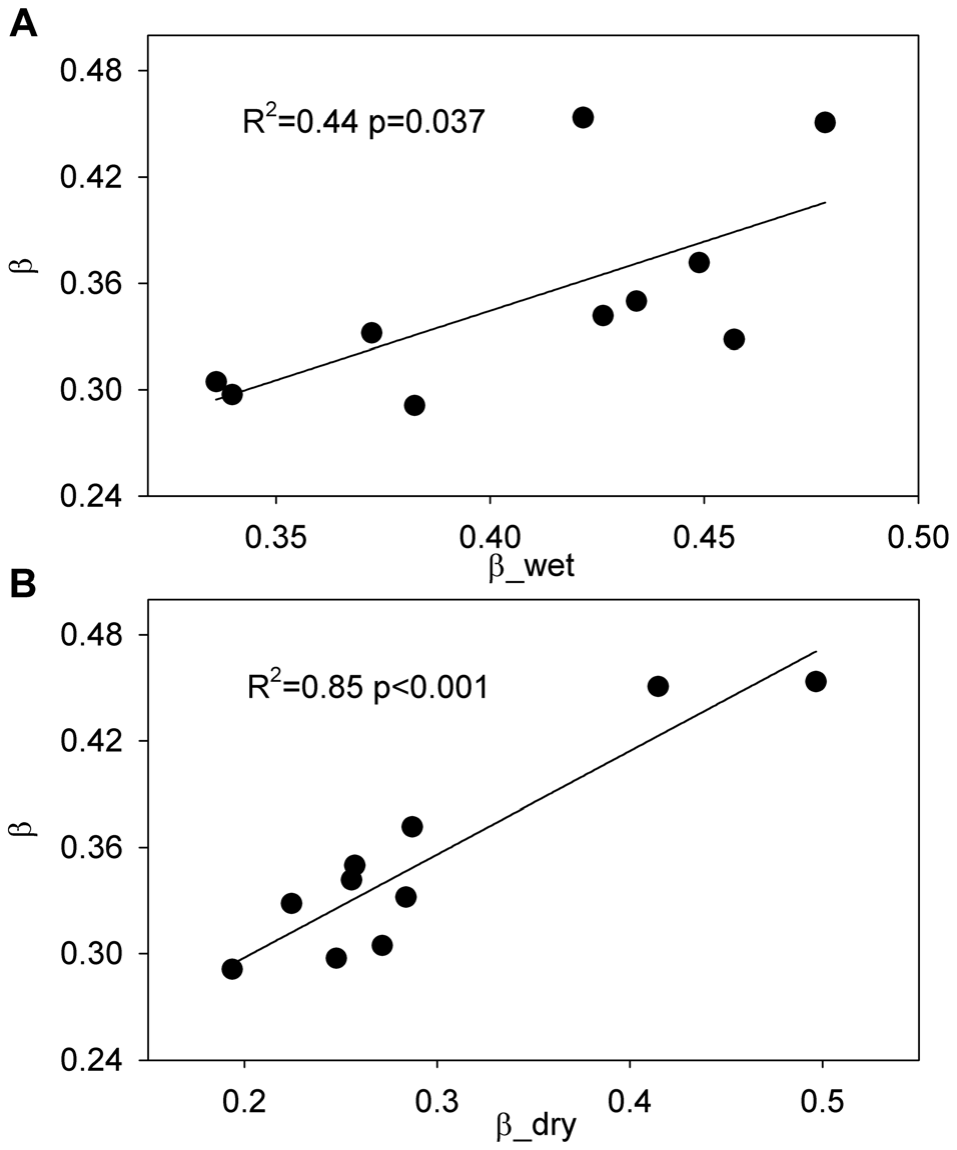
Relationships between the annual Bowen ratio (β) and Bowen ratio during wet and dry seasons. Bowen ratio during (A) the wet season (β_wet) was calculated as the ratio of sensible heat flux to latent heat flux from November to June at each year, and during (B) the dry season (β_dry) was calculated as the ratio of sensible heat flux to latent heat flux from July to October at each year, respectively.

The Bowen ratio decreased during the vigorous growth period (March-October), as the latent heat flux was higher than the sensible heat flux in these months (Fig. 3). From 2003 to 2012, because the dry season occurred during the vigorous growth period of the forest, the mean value of the Bowen ratio during the dry season (0.29±0.09) was lower than that during the wet season (0.41±0.05). The maximum (0.50) and minimum (0.11) Bowen ratio during the dry season were observed in 2003 and 2011, respectively, which correspond to the maximum (391 MJ m^−2^) and minimum (176 MJ m^−2^) sensible heat flux during the dry season. The Bowen ratio during the dry season and the wet season significantly correlated with the annual Bowen ratio, with coefficients of determination were 0.85 (p<0.001) and 0.44 (p = 0.037), respectively (Fig. 4).

### Factors Affecting the Interannual Variation of the Bowen Ratio

**Figure 5:**
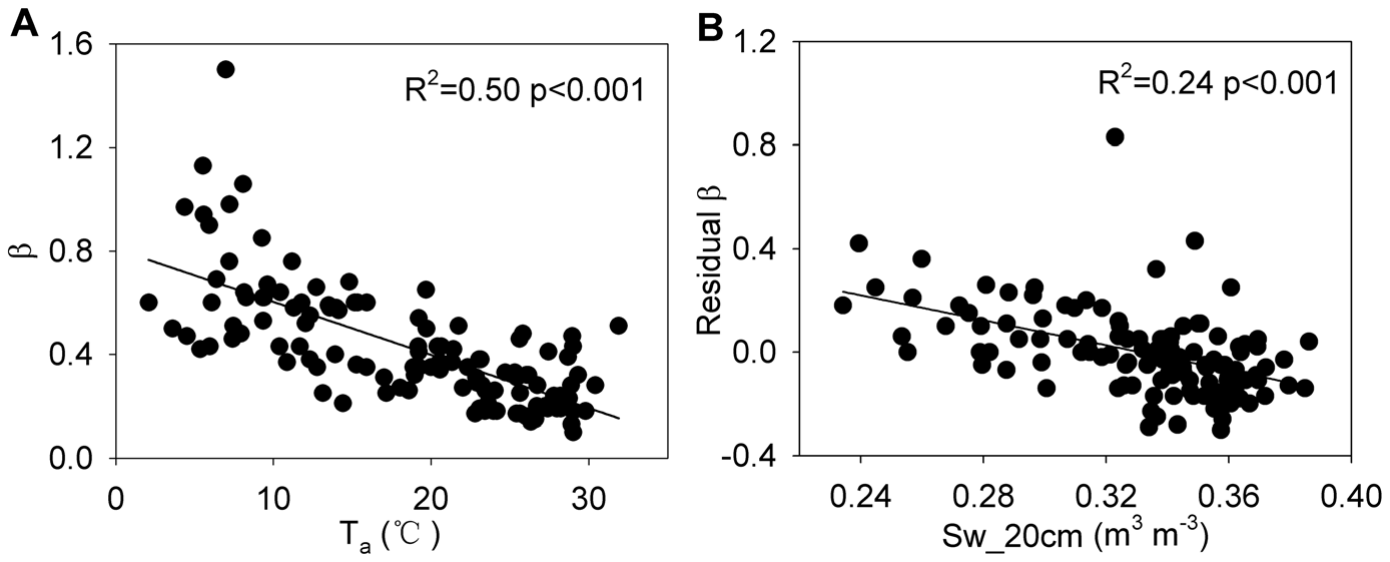
Factors affecting the Bowen ratio on a monthly timescale. The relationships (A) between the monthly Bowen ratio (β) and air temperature (T_a_), (B) between residual of the monthly Bowen ratio (β) as a function of air temperature (T_a_) and soil water content at 20 cm (Sw_20 cm).

Fig. 5 presents the dominant affecting factors for the Bowen ratio on a monthly timescale. The Bowen ratio was primarily affected by air temperature (R^2^ = 0.50, p<0.001) (Fig. 5A). The net radiation, vapor pressure deficit, soil water content at 20 and 50 cm, and enhanced vegetation index also significantly correlated with the monthly Bowen ratio, with coefficients of determination were 0.43, 0.29, 0.11, 0.22, and 0.36, respectively. While, neither the soil water content at 5 cm nor the precipitation amount significantly correlated with the monthly Bowen ratio. Removing the influence of air temperature on the monthly Bowen ratio, the residual was mainly affected by the soil water content at 20 cm (R^2^ = 0.24, p<0.001) (Fig. 5B).

**Figure 6:**
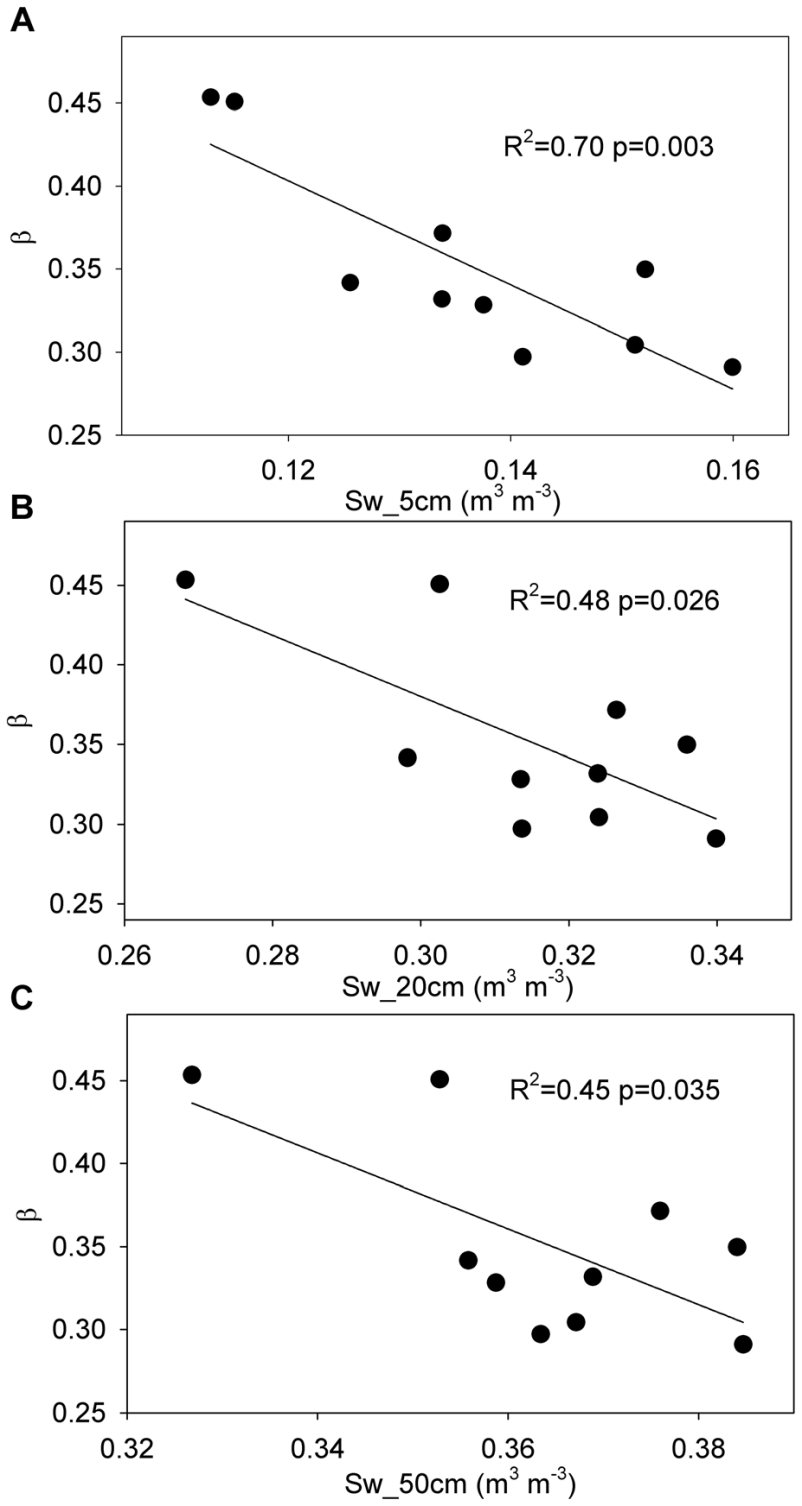
Relationships between the annual Bowen ratio (β) and dry season soil water content. Dry season (July-October) soil water content was measured at (A) 5 cm (Sw_5 cm), (B) 20 cm (Sw_20 cm), and (C) 50 cm (Sw_50 cm).

On an annual timescale, no environmental or physiological factors were observed that significantly affected the annual Bowen ratio. However, soil water content at 5 cm during the dry season played a key role in affecting the annual Bowen ratio (R^2^ = 0.70, p = 0.003), followed by the soil water content at 20 cm (R^2^ = 0.48, p = 0.026) and 50 cm (R^2^ = 0.45, p = 0.035) (Fig. 6). By removing the influence of soil water content at 5 cm on the annual Bowen ratio, there was no affecting factor for this residual. Further, the Bowen ratio during the dry season was also primarily affected by the soil water content at 5, 20, and 50 cm during the dry season, with coefficients of determination were 0.66 (p = 0.002), 0.63 (p = 0.026), and 0.63 (p = 0.036), respectively. Note that, there were no environmental and physiological factors during the wet season that significantly affected the annual Bowen ratio and the Bowen ratio during the wet season.

## Discussion

### Precipitation Amount and Frequency Affecting the Interannual Variation of the Bowen Ratio

The affecting effect of the precipitation amount to the annual Bowen ratio was diverse at specific forest sites [11]–[13]. Grünwald and Bernhofer (2007) [12] indicated that the annual Bowen ratio in a temperate spruce forest was primarily affected by annual precipitation amount. However, in a Japanese cypress forest [13], in a boreal Scots pine forest [11], and in this studied plantation, no affecting effect between the annual precipitation amount and annual Bowen ratio was found.

**Figure 7:**
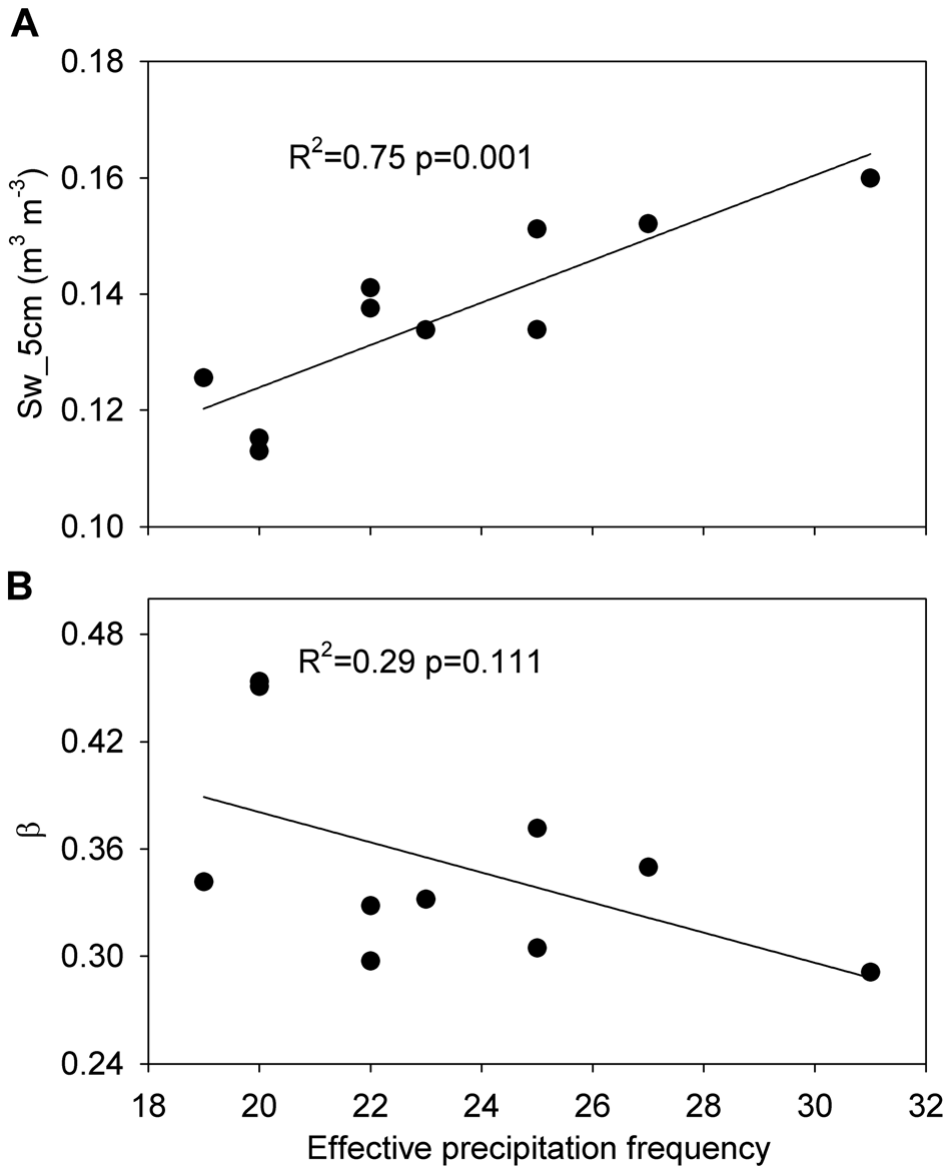
Impact of the effective precipitation frequency on soil water content and annual Bowen ratio (β). The effective precipitation frequency was measured as the number of days with a precipitation amount ≥ 1.5 mm during the dry season, and soil water content was measured at 5 cm (Sw_5 cm) during the dry season.

In this studied plantation, the annual Bowen ratio was mainly affected by the soil water content at 5 cm during the dry season (Fig. 6A). Unlike a broadleaved temperate plantation [43], a beech forest [44], and a mixed-conifer forest [45], the soil water content at 5 cm during the dry season was not significantly influenced by the precipitation amount (R^2^ = 0.38, p = 0.065). Instead, due to the precipitation interception capacity of the forest canopy [21], the soil water content at 5 cm during the dry season was significantly affected by the effective precipitation frequency (no less than 1.5 mm day^−1^) in this studied plantation (Fig. 7A). Because the maximum leaf area index was 5.6 m^2^ m^−2^ and the variation of the leaf area index was less than 1.5 m^2^ m^−2^ in this studied plantation [20], the precipitation amount of less than 1.5 mm day^−1^ was mostly intercepted by the forest canopy and did not influence the soil water content at 5 cm [21]. The results also indicated that the effective precipitation frequency during the dry season did not directly affect the annual Bowen ratio (R^2^ = 0.29, p = 0.111) (Fig. 7B). Therefore, we concluded that the effective precipitation frequency during the dry season, not the precipitation amount, indirectly affected the annual Bowen ratio through its effect on the soil water content at 5 cm during the dry season.

**Figure 8:**
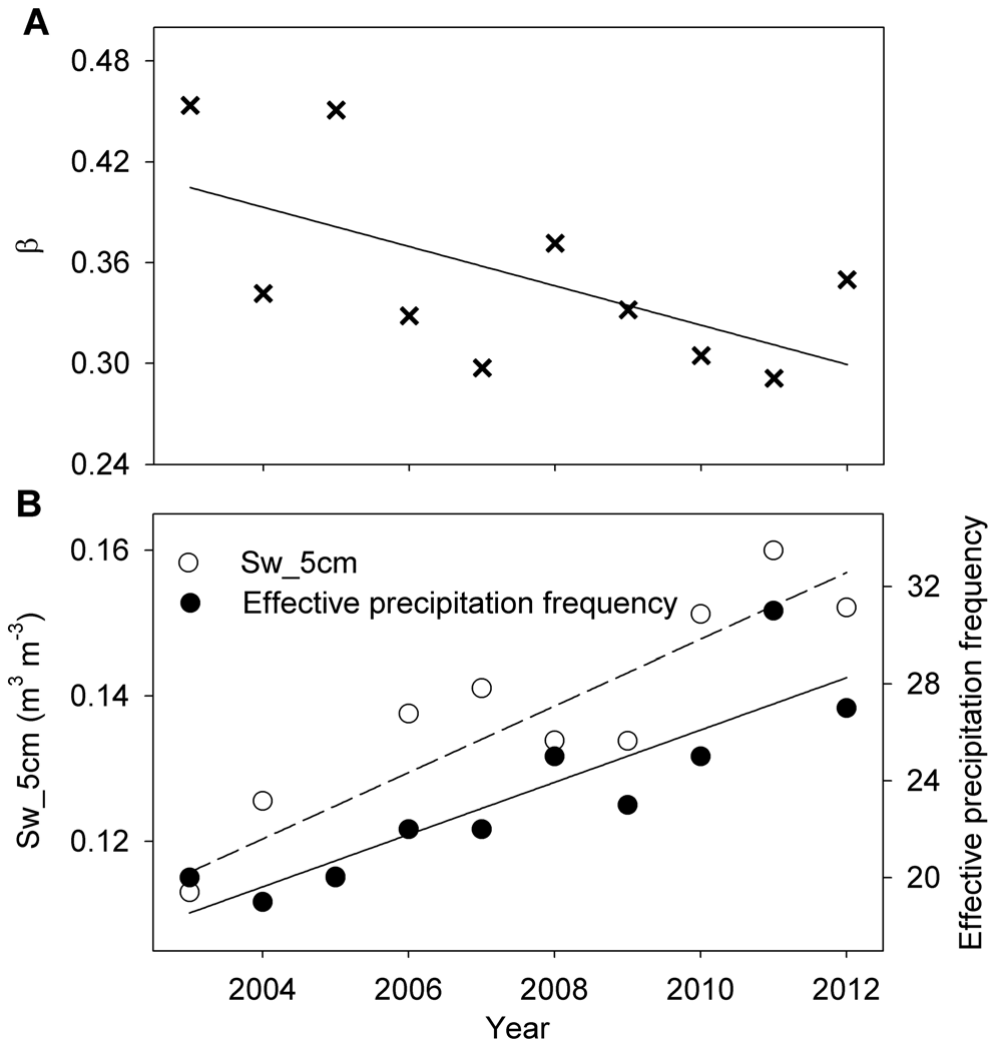
Long-term trends of the Bowen ratio (β), soil water content, and effective precipitation frequency. Long-term trends were marginally significant for (A) the annual Bowen ratio (p = 0.06), and significant for (B) the soil water content at 5 cm (Sw_5 cm; dashed line, p<0.001) and the effective precipitation frequency (solid line, p<0.001) during the dry season.

The annual Bowen ratio in this studied plantation exhibited a marginally significant decreasing trend (p = 0.061) during 2003–2012 (Fig. 8A). During the dry season, although the effective precipitation frequency did not exhibit a noticeable trend (p = 0.772) between 1985 and 2002, the effective precipitation frequency significantly increased (p<0.001) between 2003 and 2012 (Fig. 8B). The soil water content at 5 cm during the dry season also significantly increased (p<0.001) between 2003 and 2012 (Fig. 8B). Therefore, we predicted that the annual Bowen ratio will likely decrease in the future, if the effective precipitation frequency and shallow soil water content during the dry season continue to follow an increasing trend similar to that observed over the last 10 years.

### Comparison of the Bowen Ratio with Other Forests

Generally, the seasonal variation of Bowen ratio was ‘U’ shaped with the minimum value occurred in summer, particularly in temperate forests [46], [47]. While, the Bowen ratio increased during the summers of 2003, 2007, and 2010 due to severe drought stress in this studied plantation (Fig 3C). Similar to this studied plantation, the Bowen ratio increased in a ponderosa pine forest in the western United States [48] and in a deciduous broadleaved forest in the southern United States [3] due to severe drought stress. However, drought stress had no discernible impact on the Bowen ratio in a tropical rain forest in the Amazon [31]. This discrepancy could be attributed to differences in drought stress conditions and in water use strategies of forests to cope with drought stress [3], [31], [32], [49].

The annual mean Bowen ratio in this studied plantation was 0.35±0.06 during 2003–2012. This value was lower than the reported values of 0.55 in a temperate Douglas-fir [50], 0.74 in a temperate mixed forest [46], 0.81 in a boreal scots pine forest [11], 0.89 in a loblolly pine plantation [47], and was similar to 0.39 in a tropical palm forest [51]. A tropical rainforest in eastern Amazonia experienced a lower annual Bowen ratio (0.17) [31] compared to the studied plantation.

### Relationships between Latent Heat Flux and Sensible Heat Flux and Their Effect on the Bowen Ratio

Although the Bowen ratio is the relative change between the sensible heat flux and latent heat flux, the sensitivity of the Bowen ratio to the latent heat flux and sensible heat flux, and affecting factors of these fluxes are diverse [3], [11]–[13]. For example, in a temperate spruce forest [12] and a boreal Scots pine forest [11], the annual Bowen ratio was significantly correlated with the latent heat flux and sensible heat flux. In the temperate spruce forest, both the annual sensible heat flux and Bowen ratio were significantly affected by the annual precipitation amount [12]. Meanwhile, both the annual sensible heat flux and Bowen ratio were influenced by annual net radiation in the boreal Scots pine forest [11]. However, there were no factors that significantly affected the annual latent heat flux in these two forests.

In this studied plantation, the annual Bowen ratio was neither significantly correlated with the latent heat flux (R^2^ = 0.32, p = 0.087) nor correlated with sensible heat flux (R^2^ = 0.20, p = 0.193). The annual net radiation played a key role in affecting both the annual latent heat flux (R^2^ = 0.61, p = 0.008) and sensible heat flux (R^2^ = 0.59, p = 0.009). However, on an annual timescale there were no factors that significantly influenced the annual Bowen ratio. On a monthly timescale, the Bowen ratio was significantly correlated with latent heat flux (R^2^ = 0.49, p<0.001) and sensible heat flux (R^2^ = 0.03, p = 0.045). The monthly latent heat flux and sensible heat flux were mainly affected by net radiation (R^2^ = 0.86, p<0.001) and vapor pressure deficit (R^2^ = 0.36, p<0.001), respectively. However, the monthly Bowen ratio was primary influenced by air temperature (R^2^ = 0.50, p<0.001). Thus, we could not predict the factors affecting the interannual variation of the Bowen ratio by directly inferred from the affecting factors of the latent heat flux or sensible heat flux.

## Conclusions

This study demonstrated that the effective precipitation frequency during the dry season, not the precipitation amount, through its positive effect on the soil water content at 5 cm, indirectly affected the annual Bowen ratio during 2003–2012. Meanwhile, the annual Bowen ratio exhibited a marginally significant decreasing trend (p = 0.061). Assuming that the effective precipitation frequency and soil water content at 5 cm during the dry season continue to increase (p<0.001) due to climate change, as was observed during 2003–2012, the annual Bowen ratio may decrease further. Thus, the warming effect of available energy on the surface air may also decline in this studied plantation. This consequence is consistent with the studies reported by Lee et al. [52] and Wickham et al. [53]. These studies demonstrated that the warming effect of available energy on the forest surface air is weaker in mid and low latitudes than in high latitudes, due to the forest biophysical adjustment mechanisms [52], [53]. Further studies of long-term trend of Bowen ratio and its affecting mechanism are necessary to acknowledge the warming effect of available energy on the forest surface air in response to climate change.
